# Metagenomic Analysis Indicates *Epsilonproteobacteria* as a Potential Cause of Microbial Corrosion in Pipelines Injected with Bisulfite

**DOI:** 10.3389/fmicb.2016.00028

**Published:** 2016-01-28

**Authors:** Dongshan An, Xiaoli Dong, Annie An, Hyung S. Park, Marc Strous, Gerrit Voordouw

**Affiliations:** ^1^Petroleum Microbiology Research Group, Department of Biological Sciences, University of CalgaryCalgary, AB, Canada; ^2^Department of Geosciences, University of CalgaryCalgary, AB, Canada

**Keywords:** corrosion, pipeline, microbiologically influenced corrosion (MIC), microbial community analysis, metagenomics, sulfate reducing bacteria (SRB), *Epsilonproteobacteria*, hydrogenase

## Abstract

Sodium bisulfite (SBS) is used as an oxygen scavenger to decrease corrosion in pipelines transporting brackish subsurface water used in the production of bitumen by steam-assisted gravity drainage. Sequencing 16S rRNA gene amplicons has indicated that SBS addition increased the fraction of the sulfate-reducing bacteria (SRB) *Desulfomicrobium*, as well as of *Desulfocapsa*, which can also grow by disproportionating sulfite into sulfide, sulfur, and sulfate. SRB use cathodic H_2_, formed by reduction of aqueous protons at the iron surface, or use low potential electrons from iron and aqueous protons directly for sulfate reduction. In order to reveal the effects of SBS treatment in more detail, metagenomic analysis was performed with pipe-associated solids (PAS) scraped from a pipe section upstream (PAS-616P) and downstream (PAS-821TP) of the SBS injection point. A major SBS-induced change in microbial community composition and in affiliated *hynL* genes for the large subunit of [NiFe] hydrogenase was the appearance of sulfur-metabolizing *Epsilonproteobacteria* of the genera *Sulfuricurvum* and *Sulfurovum*. These are chemolithotrophs, which oxidize sulfide or sulfur with O_2_ or reduce sulfur with H_2_. Because O_2_ was absent, this class likely catalyzed reduction of sulfur (S^0^) originating from the metabolism of bisulfite with cathodic H_2_ (or low potential electrons and aqueous protons) originating from the corrosion of steel (Fe^0^). Overall this accelerates reaction of of S^0^ and Fe^0^ to form FeS, making this class a potentially powerful contributor to microbial corrosion. The PAS-821TP metagenome also had increased fractions of *Deltaproteobacteria* including the SRB *Desulfomicrobium* and *Desulfocapsa*. Altogether, SBS increased the fraction of hydrogen-utilizing *Delta-* and *Epsilonproteobacteria* in brackish-water-transporting pipelines, potentially stimulating anaerobic pipeline corrosion if dosed in excess of the intended oxygen scavenger function.

## Introduction

Pipeline failure caused by corrosion can have serious consequences for the oil and gas industry (Ossai et al., 2015) and understanding the causes of corrosion is therefore important. While chemical reaction between oxygen and iron is the main cause of external pipeline corrosion, microbiologically influenced corrosion (MIC) under mostly anoxic conditions can account for up to 40% of internal pipeline corrosion in the oil and gas industry (Zhu et al., 2003). The application of high-throughput sequencing technologies has indicated that diverse microbes are involved in internal pipeline corrosion, such as sulfate reducing bacteria (SRB), acid-producing fermentative bacteria, including acetogens, as well as methanogens (Dinh et al., 2004; Park et al., 2011; Mand et al., 2014; Okoro et al., 2014; Yang et al., 2014). Among these SRB are often considered to be the major MIC causative agents.

Fundamentally all SRB can corrode iron indirectly by producing the corrosive chemical agent hydrogen sulfide (H_2_S). This has been referred to as “chemical microbially-influenced corrosion” (CMIC) by Enning and Garrelfs (2014). H_2_S is produced during sulfate reduction by SRB with electrons usually derived from organic acids, alcohols, or hydrogen (H_2_), which is formed by fermentation of organic compounds in anoxic settings (Muyzer and Stams, 2008). In the absence of organic electron donors and in the presence of metallic iron, SRB may obtain energy from oxidation of cathodic H_2_ formed by chemical reaction between protons from water and electrons from anodic dissolution of iron, accelerating corrosion (Mand et al., 2014). Whether SRB are capable of accelerating corrosion by scavenging cathodic H_2_, which was proposed long ago (Von Wolzogen Kühr and Van der Vlugt, 1934), is still controversial (Enning and Garrelfs, 2014). Instead, some SRB are thought to corrode iron through direct uptake of the anodic electrons with protons from water for sulfate reduction (“electrical microbially-influenced corrosion”; EMIC; Enning and Garrelfs, 2014).

The microbial consumption or production of H_2_ is catalyzed by hydrogenases that can be divided into two main phylogenetically unrelated groups, the [NiFe]- and [FeFe]-hydrogenases (Vignais and Billoud, 2007). [NiFe]-hydrogenases are common in *Archaea* and *Bacteria*. Many are external to the cytoplasm and are primarily associated with H_2_ oxidation in oxic and anoxic metabolism. [FeFe]-hydrogenases are found in *Bacteria* and *Eukarya*. These enzymes are especially prevalent in the cytoplasm of anaerobic fermentative organisms (e.g., the *Firmicutes*), where they form hydrogen in metabolic reactions coupling the oxidation of reduced electron carriers (NADH, NADPH or reduced ferredoxin, Fd_red_) to the reduction of protons. SRB of the genus *Desulfovibrio* are exceptional in having a periplasmic [FeFe]-hydrogenase, which functions in hydrogen oxidation at high hydrogen concentration (Caffrey et al., 2007). Hence, assuming that consumption of cathodic hydrogen is important in MIC, both types of hydrogenases can contribute. However, [NiFe]-hydrogenases may be more important than [FeFe] hydrogenases because these enzymes are more widespread and act at lower hydrogen concentrations. Likewise, if metabolism of anodic electrons and aqueous protons in EMIC involves formation of H_2_ both types of enzymes may contribute. In addition to SRB, hydrogenotrophic methanogens and acetogens have been found to contribute to MIC by using cathodic H_2_ or anodic electrons for reduction of CO_2_ to methane and acetate, respectively (Dinh et al., 2004; Mand et al., 2014).

As indicated previously (Park et al., 2011), the presence of MIC-causing SRB can be promoted by injection of sodium bisulfite (SBS), which is used as an oxygen scavenger to decrease oxygen-mediated corrosion in pipelines and other steel infrastructure. Injection of SBS into pipelines transporting brackish subsurface water to a plant generating steam for production of bitumen by steam-assisted gravity drainage caused a drastic change in microbial community composition of pipe-associated solids (PAS). Relative to solids from a pipe section upstream of the SBS injection point (PAS-616P), solids from a downstream pipe section (PAS-821TP) had a smaller fraction of methanogens of the family *Methanobacteriaceae* and larger fractions of SRB of the genera *Desulfomicrobium* and *Desulfocapsa* (Park et al., 2011). *Desulfocapsa* can also grow by disproportionating bisulfite into sulfide and sulfate (Finster, 2008). Here we evaluate the genetic potential of the microbial communities in these two PAS samples in more detail by an in depth metagenomic analysis with a focus on hydrogenase genes.

## Materials and methods

### Sample collection

Two cutouts from a brackish water-transporting pipeline system were collected upstream (616P) and downstream (821TP) from the SBS injection point. These were the same as described elsewhere (Park et al., 2011). The pipeline cutouts were immersed in pipe-associated water (PAW) from the site, were shipped in sealed, airtight buckets and received in the lab within 24 h. The cutouts and the associated waters were then immediately transferred to a Coy anaerobic hood with an atmosphere of 90% (v/v) N_2_ and 10% CO_2_. PAS-616P and PAS-821TP were obtained by scraping the drained surface of the cutouts with a sterile spatula. These were then re-suspended in 260 mL of PAW-616P and PAW-821TP, respectively, filtered using an 0.2 μm Millipore filter (Nylon membrane, USA) prior to use.

Chemical analyses conducted on the samples included the measurement of pH, sulfide (Trüper and Schlegel, 1964), sulfate (ion chromatography with conductivity detector/anion column), ammonium, nitrite (ion chromatography with UV detector/anion column), and organic acids (ion chromatography with UV detector/organic acids column), as detailed else where (Park et al., 2011).

### DNA isolation

DNA was extracted from the PAS samples using a bead-beating procedure outlined by the manufacturer of the FastDNA® Spin Kit for Soil (MP Biomedicals). The extracted DNA was further purified by cesium chloride density gradient centrifugation. The concentration of DNA was quantified using the Qubit Fluorometer, and Quant-iT™ dsDNA HS Assay Kit (Invitrogen). A total of 20.5 and 25.8 μg of CsCl-purified DNAs were obtained from PAS-821TP and PAS-616P, respectively. The purified DNAs were then used for pyrosequencing of 16S rRNA gene (16S) amplicons and for metagenome sequencing.

### Pyrosequencing of 16S amplicons

Amplification of 16S genes was with non-barcoded 16S primers 926Fw (AAACTYAAAKGAATTGRCGG) and 1392R (ACGGGCGGTGTGTRC) in the first PCR and with FLX titanium amplicon primers 454_RL_X and 454T_FwB in the second PCR. The latter primers have the sequences for 926Fw and 1392R as their 3′ ends. Primer 454T_RL_X has a 25 nucleotide A-adaptor (CCATCTCATCCCTGCGTGTCTCCGAC) and a 10 nucleotide multiplex identifier barcode sequence X. Primer 454T_FwB has a 25 nucleotide B-adaptor sequence (CTATGCGCCTTGCCAGCCCGCTCAG). The first PCR was run for 5 min at 95°C, followed by 25 cycles of 30 s at 95°C, 45 s at 55°C, and 90 s at 72°C and finally 10 min at 72°C. The PCR products were used as templates for a second PCR of 10 cycles under the same conditions. PCR products were checked on an agarose gel and purified with a QIAquick PCR Purification kit (Qiagen). The amounts of purified 16S amplicons were then normalized to 20 μl of 20 ng/μl and sent for pyrosequencing to the Genome Quebec and McGill University Innovation Centre (Montreal, QC). Pyrosequencing was performed in a Genome Sequencer FLX Instrument, using a GS FLX Titanium Series Kit XLR70 (Roche Diagnostics Corporation). The 16S sequence reads were analyzed with Phoenix 2 (Soh et al., 2013).

### Metagenome sequencing

Metagenome sequencing was performed with both 454- and Illumina-platforms at the Genome Quebec and McGill University Innovation Centre. For the 454 sequencing, single-end shotgun DNA libraries were prepared from 1.0 μg of purified DNA and sequenced with the 454 Life Sciences GS-FLX genome sequencer using titanium chemistry and standard library construction procedures (Roche Applied Science, Laval, Quebec, Canada).

For the Illumina sequencing, DNA libraries were prepared using the TruSeq DNA Sample Prep Kit v1 (Illumina, San Diego, CA) as per the manufacturer's instructions starting with 2 μg of purified DNA. The libraries were loaded onto the flow cell, one per lane using a cBot (Illumina). Sequencing to obtain paired end 150 bp reads was performed on a HiSeq 2000 instrument (Illumina) as per the manufacturer's instructions.

### Metagenomic analysis and assembly

All 454 and Illumina metagenomic raw reads were subjected to quality control (QC) and an assembly process using the in-house developed software described by Saidi-Mehrabad et al. (2013) and Tan et al. (2013). Ribosomal RNA genes were identified with Meta-RNA (Huang et al., 2009) from 454 QC reads. All raw 454 and Illumina sequence data were submitted to the Short Read Archive under accession numbers SRX559897, SRX559898, SRX559901, and SRX559902.

### Gene abundance analysis

Genes for large hydrogenase subunits (referred to as *hydL* for [FeFe]-hydrogenase and *hynL* for [NiFe] hydrogenase) and the house keeping gene *rpoB*, the gene for the RNA polymerase β subunit, were sought in the six-frame translated metagenomic contigs using hmmsearch with a cutoff *e*-value of e-5. The *hydL* (PF02906) and *hynL* (PF00374) hidden Markov models (HMMs) were downloaded from the Pfam database (Finn et al., 2014) and the *rpoB* (TIGR02013, TIGR03670) HMMs were downloaded from TIGRFAMs (Haft et al., 2013). In the aligned region, the translated amino-acid sequences and the corresponding nucleotide sequences were extracted and included in the further gene abundance and phylogenetic analyses.

The QC metagenomic reads were mapped against the *hydL, hynL*, and *rpoB* nucleotide gene sequences using bowtie2 (Langmead and Salzberg, 2012) and the mapping files were processed using Picard with “MarkDuplicates” options (http://broadinstitute.github.io/picard/) to remove PCR duplicates. The BEDTools (Quinlan and Hall, 2010) and in-house perl scripts were then used to calculate coverage information for the extracted genes. The coverages were subsequently normalized to the length of the respective genes to make sure that longer genes did not have higher coverage values just because of their length. The length-normalized coverages were then further normalized to the total number of *rpoB* genes in the metagenomic samples, yielding an approximation of the number of genes per genome for each extracted hydrogenase genes.

### Phylogenetic analysis

Phylogenetic trees were constructed for *hynL* genes obtained from the PAS-616P and PAS-821TP metagenomes with a multi-step approach using reference alignments and trees in order to minimize errors and biases introduced by the fragmentary and non-overlapping nature of the metagenomic sequences (Brazelton et al., 2012). The reference multiple sequence alignment was constructed from multiple sequence alignments generated in other work (Vignais et al., 2001) by using mafft version 7.245 with “–merge” option (Katoh and Standley, 2013). Unaligned metagenomic fragments were added to the reference alignment profile using mafft with “–add” option in order to avoid alteration of the relative positions of residues in the reference alignment. Next, a bootstrapped maximum-likelihood tree was constructed from the reference-only alignment using the “-f a” algorithm in RAxML version 8.2.3 (Stamatakis, 2014). The reference-only tree with the highest-likelihood was used as a constraint tree (“-r” flag in RAxML) for 100 inferences from the full alignment (including metagenomic fragments) by RAxML, and bootstrap support values were drawn on the highest-likelihood tree (Brazelton et al., 2012).

## Results

### Chemical characteristics of pipe samples

The pipe associated water (PAW) of the 616P and 821TP cutouts had a neutral pH and a sulfate concentration of 0.01 mM (Table S1). No sulfide was observed in either water sample. Higher concentrations of sulfate were observed in suspended PAS samples PAS-616P (0.33 mM) and PAS-821TP (0.09 mM). Passage through the SBS injection point increased the sulfide concentration from 0 mM in PAS-616P to 90 mM in PAS-821TP and the ferrous iron concentration from 1.0 mM in PAS-616P to 1080 mM in PAS-821TP (Table S1). Acetate (0.85 mM), butyrate (0.20 mM), and propionate (0.17 mM) were detected in PAW-616P, but not in PAS-616P. Acetate (0.80 mM) and propionate (0.10 mM) were also detected in PAS-821TP, but not in PAW-821TP (Table S1). These data indicate that the chemical compositions of PAS-616P and PAS-821TP were different (reflecting SBS injection) and that these differed in turn from those of the corresponding planktonic samples.

### Community compositions from 16S amplicon sequencing

The 454 pyrosequencing platform produced 20,451 good 16S reads for the two PAS samples (Table 1). These were grouped into 141 operational taxonomic units (OTUs) at a sequence dissimilarity cut-off of 5%. The average Good's coverage of 99.7% suggested that the majority of the phylotypes present in the samples had been identified. A total of 76 taxa was found in the two samples. The microbial community diversity of PAS-616P was slightly lower than that of PAS-821TP, as reflected by the estimated OTUs (Chao) and Shannon index (Table 1). PAS-616P had 75% *Archaea* and 20% *Deltaproteobacteria*, whereas PAS-821TP had 50% *Archaea* and 46% *Deltaproteobacteria* (Table 1). The increase in *Deltaproteobacteria*, ferrous iron, and sulfide from PAS-616P to PAS-821TP, likely resulted from SBS injection.

**Table 1:** **Summary of 16S amplicon sequence data and derived diversity parameters for CsCl-purified DNAs from PAS samples also used for metagenomic analysis**.

**Sample name**	**Number of QC reads^a^**	**Archaea (%)**	**Delta^b^ (%)**	**Observed OTUs (5% cutoff)**	**Estimated OTUs (Chao)^c^**	**Shannon's H index^c^**	**Good's coverage (%)**	**Number of taxa**
PAS-616P	10,772	75	20	99	128	1.28	99.7	59
PAS-821TP	9679	50	46	96	155	1.44	99.6	53
Combined	20,451	63	32	141	ND	ND	99.7	76

a *The accession numbers for the PAS-616P and PAS-821TP reads are SRX1427962 and SRX1430039, respectively*.

b *Delta is Deltaproteobacteria*.

c *Diversity indices were calculated using a normalized number of 9679 reads*.

Methanogenic archaea can contribute to MIC in anoxic pipeline systems together with SRB and acetogens. Methanogenic orders in the two PAS samples included the hydrogenotrophic *Methanobacteriales* and *Methanomicrobiales*, as well as the *Methanosarcinales*. This included the *Methanobacteriaceae* (68.5 and 48.2%), *Methanocalculus* (0.05 and 0.01%), and the methylotrophic genus *Methanolobus* (6.4 and 1.6%) in PAS-616P to PAS-821TP, respectively (Table S2).

**Figure 1:**
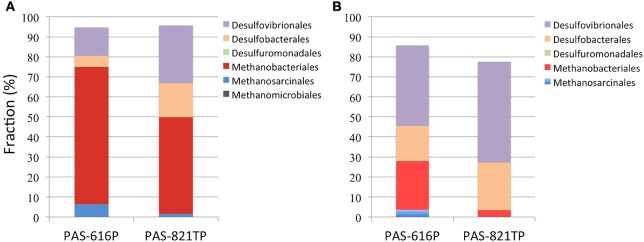
**Order-level composition of ***Archaea*** and ***Deltaproteobacteria*** derived from (A) 16S amplicons and (B) 16S sequences of 454 metagenomes**. The deltaproteobacterial orders *Myxococcales* and *Syntrophobacterales* are not obvious due to their low abundance in both libraries. The increased fractions of archaeal orders in **(A)** are likely caused by PCR bias.

Five *Deltaproteobacterial* orders, *Desulfovibrionales, Desulfobacterales, Desulfuromonadales, Myxococcales*, and *Syntrophobacterales*, were detected in the 16S amplicons (Figure 1A). Of these the first two represent potential SRB. *Desulfomicrobium* was the dominant genus within the *Desulfovibrionales* with proportions of 14.1 and 28.9% in PAS-616P and PAS-821TP amplicon libraries, respectively (Table S2). *Desulfomicrobium* species use hydrogen or simple organic compounds, including formate, ethanol, lactate, pyruvate, malate, or fumarate, as electron donors for sulfate respiration. However, in the absence of sulfate they grow fermentatively on some of these same organic substrates (Rabus et al., 2015). Within the *Desulfobacterales, Desulfocapsa* was the dominant taxon with proportions of 5.3 and 16.3% in PAS-616P and PAS-821TP, respectively (Table S2). A unique feature of this genus is its ability to grow by disproportionation of thiosulfate, sulfite, or sulfur (in the presence of ferric iron) in the absence of molecular hydrogen. In the presence of molecular hydrogen, reduction of sulfur and of sulfur oxyanions dominates over disproportionation (Finster, 2008). *Desulfuromonadales* of the genus *Desulfuromonas* were detected at 0.14 and 0.01% in the PAS-616P and PAS-821TP amplicons, respectively (Table S1).

Hence a survey of the 16S amplicons of the CsCl-purified DNAs confirmed earlier findings (Park et al., 2011), which indicated a decrease in methanogenic taxa and an increase in sulfate- and sulfite-reducing and sulfite-disproportionating taxa when passing from upstream to downstream of the SBS injection site.

### Community compositions from metagenomic 16S genes

Metagenome sequencing of the PAS samples was performed using both 454 and Illumina platforms. Because of the limitation of fragment sizes in Illumina sequencing, only the 454 16S rRNA gene sequences were used for the analysis of taxonomic profiles of the two metagenomes. A total of 270,379 and 337,937 reads with mean lengths of 659 and 654 bp were obtained for the PAS-616P and PAS-821TP metagenomes, respectively (Table 2). Among these 470 and 703 reads were identified as 16S fragments in the PAS-616P and PAS-821TP metagenomes (Table 2). This corresponds to one 16S read per 575 and 480 total reads, respectively. Taxonomic assignments of these 16S reads revealed that *Deltaproteobacteria* (58.3%) and *Archaea* (27.9%) dominated in the PAS-616P metagenome (Table 2). A larger fraction of *Deltaproteobacteria* (76.1%) and smaller fraction of *Archaea* (3.4%) was found in the PAS-821TP metagenome (Table 2). Hence these changed similarly as observed for the 16S amplicon libraries (Table 2).

**Table 2:** **Summary of 454 metagenomic data obtained for CsCl purified DNAs from the PAS samples**.

**Parameter**	**Metagenome**		**16S amplicon**	
	**PAS-616P**	**PAS-821TP**	**PAS-616P**	**PAS-821TP**
Reads	270,379	337,937	10,772	9679
16S rRNA (>180 bp)	470	703	10,772	9679
*Archaea*	27.87	3.41	74.99	49.86
*Bacteria*	72.13	93.88	25.01	50.14
*Actinobacteria*	0.85	0	0.31	0.05
*Bacteroidetes*	0.85	1.00	0.21	0.37
*Chloroflexi*	5.32	3.56	1.61	1.44
*Firmicutes*	1.06	1.56	0.21	0.07
*Deltaproteobacteria*	58.30	76.10	19.84	45.90
*Epsilonproteobacteria*	0.64	5.55	0.02	0.19
*Spirochaetes*	0.21	0.28	0.07	0.27

*Fractions (%) of taxa at the kingdom, phylum or class level from metagenomic 16S reads are compared with those obtained from 16S amplicon sequencing*.

*Methanobacteriales* (24.3%) and *Methanosarcinales* (3.6%) were the dominant methanogenic orders in the PAS-616P metagenome, while only *Methanobacteriales* (3.4%) were identified in the PAS-821TP metagenome (Figure 1). These included the *Methanobacteriaceae* and the genus *Methanolobus* (Table S2). The SRB were represented by the orders *Desulfovibrionales* (40.2%), *Desulfobacterales* (17.4%), and *Desulfuromonadales* (0.2%) in the PAS-616P metagenome, whereas only *Desulfovibrionales* (50.4%) and *Desulfobacterales* (23.8%) were detected in the PAS-821TP metagenome (Figure 1B). *Desulfomicrobium* and *Desulfocapsa* were the dominant genera in the orders *Desulfovibrionales* and *Desulfobacterales* in these two metagenomes (Table S2).

The frequency of 16S genes representing *Epsilonproteobacteria* increased from the PAS-616P to the PAS-821TP metagenome from 0.64 to 5.6% (Table 2). This increase was also seen in the 16S amplicon libraries (Table 2: from 0.02 to 0.19%). The smaller values of these fractions reflect the fact that the primers used did not optimally amplify this class (An et al., 2013). The *Epsilonproteobacteria* consisted mainly of the genera *Sulfuricurvum* (0.43%) and *Arcobacter* (0.21%) in the PAS-616P metagenome and of *Sulfuricurvum* (4.4%) and *Sulfurovum* (0.85%) in the PAS-821TP metagenome (Table S2). *Sulfuricurvum* and *Sulfurovum* are known to oxidize sulfide to sulfate (Kodama and Watanabe, 2004). This group of mainly chemolithotrophic bacteria may also reduce sulfur (S^0^) to sulfide using H_2_ as the electron donor (Gevertz et al., 2000).

### Abundance of hydrogenase genes in PAS metagenomes

Determining the prevalence of the *hynL* and *hydL* genes for the large subunits of [NiFe]- and [FeFe]-hydrogenase indicates the genetic potential of microbial communities for consuming or producing H_2_. In order to assess the effect of SBS treatment in the brackish water-transporting pipeline system on this potential, we performed hmmsearches for potential homologs of the *hynL* and *hydL* genes against contigs of the merged 454 and Illumina assemblies of the PAS-616P and PAS-821TP metagenomes. The aligned region of assembled contigs was extracted and the unassembled metagenomic reads that passed the QC were then mapped. To get the abundance profile of either hydrogenase gene, the total length of the mapped reads was calculated and normalized by the length of the respective genes. For comparative purposes, the length-normalized coverage was further normalized by dividing by the total number of *rpoB* genes (43,059 in the PAS-616P and 35,808 in the PAS-821TP metagenomes), yielding the fraction of genes per genome (abundance) for each of the extracted hydrogenase gene types (Table 3). The hydrogenase gene sequences, obtained from hmmsearches, were taxonomically identified as the best hit in blastp searches of the NCBI database. The phylogenetic association of these genes is also given as relative abundance (Table 3: %).

**Table 3:** **Taxonomic origin and abundance of ***hydL*** genes for [FeFe]-hydrogenase and of ***hynL*** genes for [NiFe]-hydrogenase in the PAS metagenomes**.

**Gene and taxon**	**PAS-616P**	**(%)**	**PAS-821TP**	**(%)**
*hydL* for [FeFe]-hydrogenase	0.044	100.00	0.057	100.00
*Bacteroidetes*	0.003	7.49	0.011	19.10
*Deltaproteobacteria*	0.006	13.04	0.006	10.68
*Firmicutes*	0.028	64.35	0.029	50.88
*Spirochaetes*	0.002	3.92	0.004	6.31
*hynL* for [NiFe]-hydrogenase	0.505	100.00	0.532	100.00
*Actinobacteria*	0.008	1.68	0.002	0.34
*Archaea*	0.111	21.98	0.039	7.38
*Bacteroidetes*	0.008	1.52	0.010	1.87
*Betaproteobacteria*	0.008	1.68	0.001	0.28
*Chloroflexi*	0.014	2.72	0.007	1.36
*Deltaproteobacteria*	0.279	55.19	0.321	60.45
*Epsilonproteobacteria*	0.000	0.06	0.041	7.63
*Firmicutes*	0.001	0.24	0.003	0.57
*Gammaproteobacteria*	0.013	2.51	0.011	2.16

*The numbers are fractions relative to those for rpoB^1^. The fractions (%) of the total of these are also given*.

1 *A total of 43,059 and 35,808 rpoB gene fragments were detected in the merged 454 and Illumina assemblies*.

The PAS-616P and PAS-821TP metagenomes for samples taken upstream and downstream of the SBS injection point in the pipeline system, respectively, had a 10-fold higher abundance of *hynL* genes for [NiFe]-hydrogenase (Table 3: 0.505 and 0.532) than of *hydL* genes for [FeFe]-hydrogenase (0.044 and 0.057). The abundance of both types of hydrogenase genes was similar in the PAS-616P and PAS-821TP metagenomes (Table 3). The phylogenetic association of *hydL* genes was similar with most (Table 3: 64 and 51%) belonging to the *Firmicutes*, consistent with a function in H_2_ production in both metagenomes. In contrast, archaeal *hynL* genes decreased from 22.0 to 7.3%, whereas deltaproteobacterial *hynL* genes increased from 55.2 to 60.4% in transitioning from the PAS-626P to the PAS-821TP metagenome (Table 3). These changes were similar to those observed for metagenome-derived 16S (Table 2). Bisulfite injection caused a large increase in *hynL* genes affiliated with *Epsilonproteobacteria* from 0.06 to 7.63% (Table 3), matching the increase in metagenome-derived 16S (from 0.64 to 5.6%). This indicates this class to have a strong potential for H_2_ oxidation.

### Phylogeny and functional diversity of *hynL* genes

**Figure 2:**
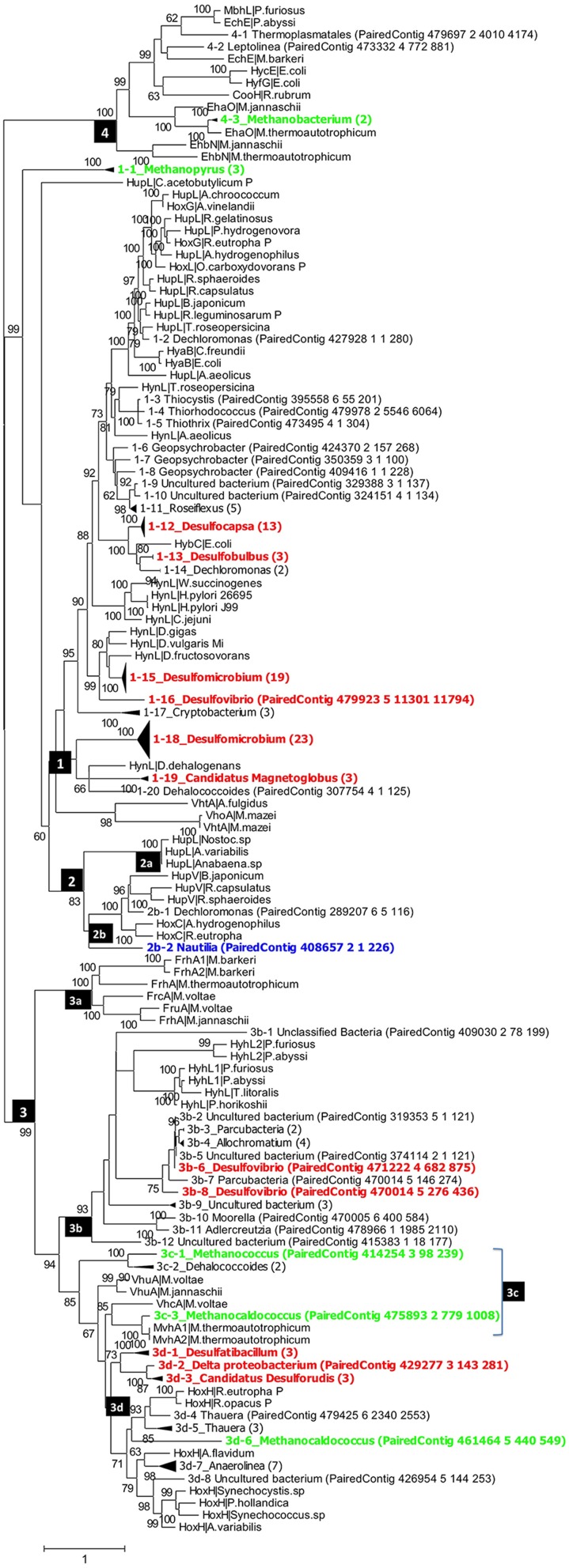
**Phylogenetic analysis of putative ***hynL*** genes for the large subunit of [NiFe]-hydrogenase detected in the PAS-616P metagenome**. Contigs with high similarity and the same taxonomic origin were collapsed into, the same clade and the number of contigs in each clade is indicated in brackets. Clades for putative methanogens, SRB, and chemolithoautotrophic SMB are highlighted in green, red, and blue. Maximum-likelihood bootstrap support values of more than 60 are shown. The numbers in the black boxes are different types of [NiFe] hydrogenases, identified by Vignais et al. (2001) as 1, 2 (2a, 2b), 3 (3a, 3b, 3c, 3d), and 4.

**Figure 3:**
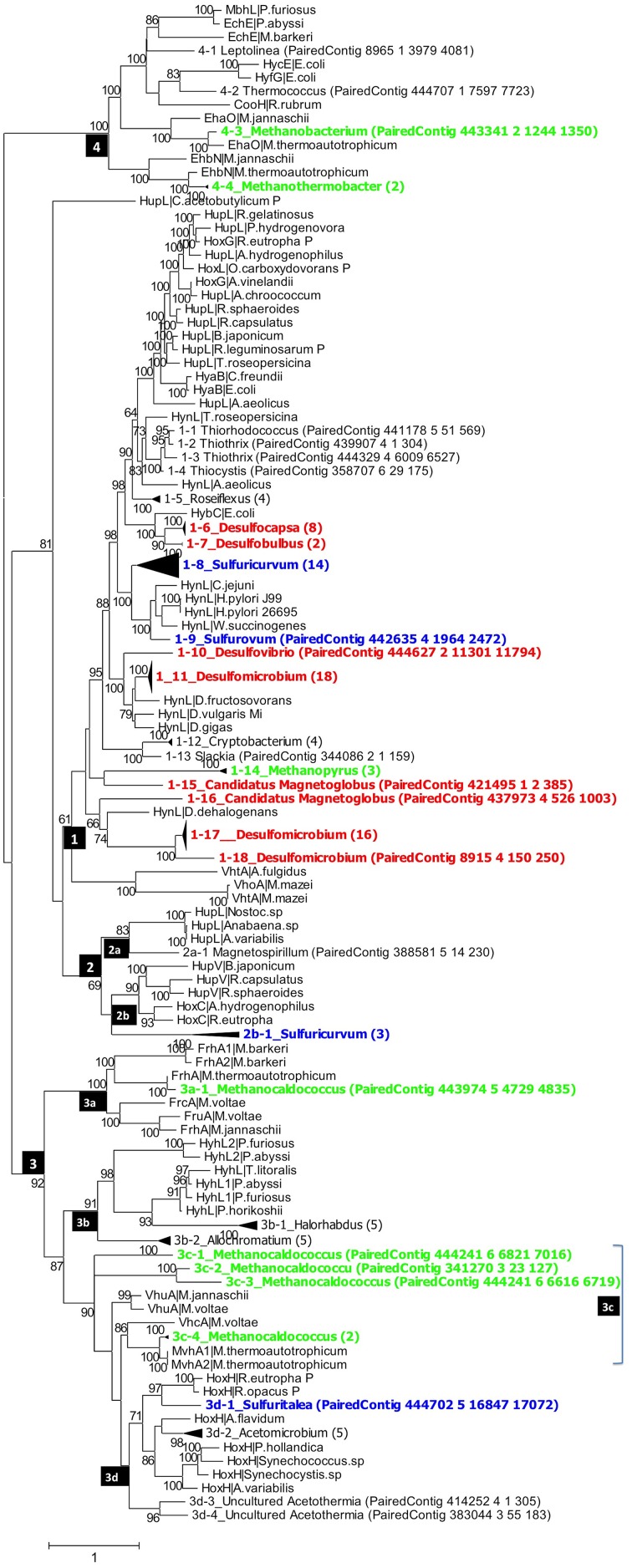
**Phylogenetic analysis of putative ***hynL*** genes for the large subunit of [NiFe]-hydrogenase detected in the PAS-821TP metagenome**. Contigs with high similarity and same taxonomic origin were collapsed into the same clade and the number of contigs in each clade is indicated in brackets. Clades for putative methanogens, SRB, and chemolithoautotrophic SMB are highlighted in green, red, and blue. Maximum-likelihood bootstrap support values of more than 60 are shown. The numbers in the black boxes are different types of [NiFe] hydrogenases, identified by Vignais et al. (2001) as 1, 2 (2a, 2b), 3 (3a, 3b, 3c, 3d), and 4.

*hynL* genes for [NiFe]-hydrogenase detected in the PAS metagenome assemblies were compared with nearest homolog sequences from the literature in phylogenetic trees, in which genes with high similarity were collapsed into the same clade. Four groups of *hynL* genes, representing [NiFe]-hydrogenases with distinct physiological roles (Vignais et al., 2001), were all observed in the PAS-616P and PAS-821TP trees (Figures 2, 3). Their phylogenetic distribution among *Deltaproteobacteria* (mostly SRB), *Archaea* (mostly methanogens), and *Epsilonproteobacteria* (mostly sulfur-metabolizing bacteria; SMB) is indicated in Table S3.

Group_1 includes the H_2_-oxidizing membrane-bound [NiFe] hydrogenases, most of which leave protons on the external side of the membrane while transporting electrons to an internally located *b*-type cytochrome. Group_1 [NiFe] hydrogenases were represented in the PAS-616PL tree in clades 1-1 to 1-20 (Figure 2). Nine of these harbored multiple contigs (indicated in brackets) with clades 1-12_Desulfocapsa_(13), 1-15_Desulfomicrobium_(19), and 1-18_Desulfomicrobium_(23) being most heavily populated. The abundance (the number of sequences divided by 43,059 observed *rpoB* sequences) of Group_1 clades was 0.365 with *Deltaproteobacteria hynL* genes (0.283) outnumbering those for *Archaea* (0.062). No Group_1 *hynL* genes for *Epsilonproteobacteria* were observed (Table S3). Group_1 *hynL* genes were represented in the PAS-821TP tree in clades 1-1 to 1-18 of which eight harbored multiple contigs (Figure 3), including 1-6_Desulfocapsa_(8), 1-7_Desulfobulbus_(2), 1-11_Desulfomicrobium_(18), and 1-17_Desulfomicrobium_(16) for the *Deltaproteobacteria*, 1-14_Methanopyrus_(3) for the *Archaea* and 1-8_Sulfuricurvum_(14) for the *Epsilonproteobacteria*. Relative to the PAS-616P metagenome, the PAS-821TP metagenome had a decreased abundance of *hynL* genes from *Archaea* (Table S3: 0.018) and an increased abundance of *hynL* genes from *Delta-* and *Epsilonproteobacteria* (Table S3: 0.363 and 0.034). The latter included *Sulfurovum*, in addition to the more dominant *Sulfuricurvum*. Filamentous *Gammaproteobacteria*, potentially oxidizing sulfide or sulfur (Chernousova et al., 2009; van der Meer et al., 2010), were observed in both metagenomes, i.e., 1-2_Thiothrix and 1-3_Thiothrix in the PAS-821TP metagenome. Hence, the continuous injection of bisulfite decreased the presence of archaeal *hynL* genes, while increasing *hynL* genes for sulfur-metabolizing *Epsilonproteobacteria*, which may use Group_1 [NiFe]-hydrogenases to oxidize H_2_ for reduction of sulfur (Campbell et al., 2006; Peters et al., 2015).

Group_2a including cyanobacterial [NiFe]-hydrogenases (Peters et al., 2015) was not significantly present. Group_2b includes regulatory [NiFe] hydrogenases, which can induce expression of *hyn* genes, when H_2_ is present. These were represented in a single clade 2b-1_Sulfuricurvum_(3) in the PAS-821TP metagenome, suggesting that expression of Group_1 *hyn* genes in *Epsilonproteobacteria* is regulated. The abundance of regulatory Group_2b *hynL* genes was lower than of Group_1 *hynL* genes (Table S3: 0.006 and 0.036, respectively).

Group_3a [NiFe]-hydrogenases are cytoplasmic enzymes involved in reduction of coenzyme F420 in methanogenic *Archaea*. This group was found in low abundance in the PAS-821TP metagenome (Table S3). Group_3b [NiFe]-hydrogenases had a diverse phylogenetic distribution, which included the SMB *Allochromatium* (both metagenomes) and the SRB *Desulfovibrio* (PAS-616P only). Group_3c includes methyl viologen-reducing [NiFe]-hydrogenases (MvhAgD), which function in the reduction of heterodisulfide with H_2_ in methanogens (Peters et al., 2015). This group included *hynL* genes from *Methanocaldococcus* (Figures 2, 3) and was more abundant in the PAS-616P than in the PAS-821TP metagenome (Table S3: 0.026 and 0.012, respectively). Group_3d includes bidirectional heteromultimeric [NiFe]-hydrogenases (HoxHY), which associate with NADH dehydrogenase for energy generation in aerobic bacteria (Peters et al., 2015). The *hydL* genes from the *Betaproteobacteria Sulfuritalea* in the PAS-821TP metagenome also belonged to this group (Figure 3: 3d-1_Sulfuritalea). In the PAS-616P metagenome Group_3d included *Firmicutes*, such as *Candidatus Desulforudis*.

The [NiFe] hydrogenases included in group 4 are typically responsible for cytoplasmic, flavodoxin-dependent H_2_ production in *Bacteria*, and methanogenic *Archaea*. The latter were represented through clade 4-3_Methanobacterium in both metagenomes. The abundance of Group_4 *hynL* genes of the genus *Methanobacterium* decreased in transitioning from the PAS-616P to the PAS-821TP metagenome (Table S3). This genus includes hydrogenotrophic methanogens with potential for iron corrosion (Dinh et al., 2004).

## Discussion

The continuous injection of bisulfite into a pipeline system transporting brackish water with little sulfate (Table 1), but high concentrations of bicarbonate (0.5–1.5 g/L; Park et al., 2011) had a significant effect on microbial community composition of the pipe wall. Analysis of metagenomic 16S reads, obtained by 454 sequencing, indicated that SBS decreased the fraction of methanogenic *Archaea* (*Methanobacteriaceae* and *Methanolobus*), but increased the fraction of *Deltaproteobacteria* (*Desulfomicrobium* and *Desulfocapsa*) and *Epsilonproteobacteria* (*Sulfuricurvum* and *Sulfurovum*), as indicated in Table 2 and Table S2.

The more extensive dataset on the distribution of *hydL* and *hynL* genes in metagenomic reads, obtained by 454 and Illumina sequencing, confirmed the shift in taxonomic distribution seen in 454 16S reads while allowing more detailed assigment of taxa involved in H_2_ metabolism. First of all *hydL* genes for [FeFe]-hydrogenase were approximately ten-fold less frequent than *hynL* genes for [NiFe] hydrogenases. Most *hydL* genes were affiliated with *Firmicutes* (Table 3), which were only a minor fraction (Table 2: 1.1–1.6%) in both metagenomes. We focused, therefore, on analysis of the phylogenetic distribution of *hynL* genes. This includes *Archaea*, which have *hynL* but do not have *hydL* genes, but may exclude *Firmicutes*, i.e., only 0.24–0.57% of all *hynL* genes was affiliated with this phylum.

Injection of bisulfite decreased the abundance of *hynL* genes from methanogenic *Archaea* of Group_1 (0.062–0.018, *Methanopyrus* in both metagenomes), Group_3c (0.026–0.012) and Group_4 (0.023–0.006), while increasing the abundance of *hynL* genes from *Deltaproteobacteria* of Group_1 (0.283–0.363), but not of Group_3b (0.006–0) and Group_3d (0.020–0). Group_1 *hynL* genes were mostly affiliated with the genera *Desulfomicrobium* and *Desulfocapsa* in both metagenomes. Other Group_1 *hynL* genes found in both metagenomes included those from *Candidatus Magnetoglobus*, which are affiliated with the *Deltaproteobacteria* class and may also be SRB (Abreu et al., 2007). Group_1 *hynL* genes from *Dechloromonas* and *Dehalococcoides*, which use H_2_ for inorganic or organic dechlorination, disappeared upon bisulfite injection (Figures 2, 3).

Injection of bisulfite strongly increased the abundance of Group_1 *hynL* genes from the *Epsilonproteobacteria Sulfuricurvum* and *Sulfurovum* (from 0 to 0.034) and of Group_2b *hynL* genes from *Sulfuricurvum*. This class also includes the phylogenetically related genera *Sulfurimonas, Sulfurospirillum, Campylobacter*, and *Arcobacter*, as well as the more distantly related *Nautilia* (Han et al., 2012). Most *Epsilonproteobacteria* are chemolithoautotrophs, which oxidize reduced sulfur species (sulfide, sulfur, and thiosulfate) with O_2_ (under microaerophilic conditions) and nitrate (Kodama and Watanabe, 2004; Campbell et al., 2006). Many also reduce sulfur with H_2_ (Campbell et al., 2006), a reaction first documented for *Arcobacter* strain FWKO_B (Gevertz et al., 2000). This is in contrast to *Betaproteobacteria* of the genus *Sulfuritalea* (Figure 3: 3d-1), which oxidize both H_2_ and reduced sulfur species with O_2_ and/or nitrate, but do not reduce sulfur with H_2_ (Kojima and Fukui, 2010). Because O_2_ is absent following SBS injection, Group_1 [NiFe]-hydrogenase of the *Epsilonproteobacteria* likely functioned in reduction of sulfur with H_2_ with the Group_2b regulatory proteins ensuring that the enzyme is expressed under these conditions. The Group_2b HoxBC [NiFe] hydrogenase sensor was first discovered in *Alcaligenes eutrophus* (now *Ralstonia eutrophus*), where HoxBC functions in H_2_ recognition and transmits the signal to HoxJ, which modulates response regulator HoxA to activate transcription of Group_1 *hyn* genes (Lenz and Friedrich, 1998).

As indicated previously (Park et al., 2011), injection of bisulfite removes O_2_ by chemical reaction:

(1) $$\begin{array}{ll} {\text{HSO}}_{3}^{-}+½{\text{O}}_{2}\to {\text{H}}^{+}+{\text{SO}}_{4}^{2-} & \; \end{array}$$

Both bisulfite (when dosed in excess) and sulfate can be reduced by SRB of the genus *Desulfomicrobium*, using cathodic H_2_ (or low potential electrons) from steel (Fe^0^) as the electron donor:

(2) $$\begin{array}{l} {\text{Fe}}^{0}+2{\text{H}}^{+}\to {\text{Fe}}^{2+}+{\text{H}}_{2} \end{array}$$

(3) $$\begin{array}{l} {\text{HSO}}_{3}^{-}+3{\text{H}}_{2}\to {\text{HS}}^{-}+3{\text{H}}_{2}\text{O} \end{array}$$

(4) $$\begin{array}{l} {\text{SO}}_{4}^{2-}+{\text{H}}^{+}+4{\text{H}}_{2}\to {\text{HS}}^{-}+4{\text{H}}_{2}\text{O} \end{array}$$

Alternatively, ${\text{HSO}}_{3}^{-}$ is disproportionated into sulfide or sulfur and sulfate by *Deltaproteobacteria* of the genus *Desulfocapsa*:

(5) $$\begin{array}{l} 4{\text{HSO}}_{3}^{-}\to {\text{HS}}^{-}+3{\text{SO}}_{4}^{2-}+3{\text{H}}^{+} \end{array}$$

(6) $$\begin{array}{l} 3{\text{HSO}}_{3}^{-}\to {\text{S}}^{0}+2{\text{SO}}_{4}^{2-}+{\text{H}}_{2}\text{O}+{\text{H}}^{+} \end{array}$$

Assuming little H_2_ production by fermentation of organics, the balance between reduction (Reactions 2 and 3) and disproportionation (Reactions 5 and 6) will depend on the availability of cathodic hydrogen (or of low potential electrons in an EMIC scenario). The generated sulfur (S^0^) is then used by *Epsilonproteobacteria*, which were clearly stimulated by injection of bisulfite and which may also use cathodic H_2_:

(7) $$\begin{array}{ll} {\text{S}}^{0}+{\text{H}}_{2}\to {\text{HS}}^{-}+{\text{H}}^{+} & \; \end{array}$$

Reactions (2) and (7) can be combined with formation of FeS from Fe^2+^ and HS^−^ to:

(8) $$\begin{array}{ll} {\text{Fe}}^{0}+{\text{S}}^{0}\to \text{FeS} & \; \end{array}$$

which indicates that *Epsilonproteobacteria* may gain energy for growth by accelerating the reaction between metallic iron and elemental sulfur, which would take place more slowly in their absence. Both FeS and S^0^ were significant components of the pipeline scale downstream from the SBS injection point (Park et al., 2011). The transported brackish water had little organic carbon (Table S1; Park et al., 2011), which could have served as an alternative electron donor for sulfur reduction. Indeed, *Deltaproteobacteria* of the genus *Desulfuromonas*, which specialize in this activity, were found in low fractions (Table S2: up to 0.14%) and decreased upon SBS injection.

Hence, the metagenomic studies presented here have uncovered a potential role for *Epsilonproteobacteria* in MIC in pipelines subjected to injection of bisulfite for the scavenging of oxygen, which is commonly used. Clearly injection of excess bisulfite should be avoided and the emergence of *Epsilonproteobacteria*, which were nearly absent upstream from the SBS injection point, may serve as an indicator of increased MIC threat.

## Author contributions

DA: Contributed to design of the study and isolation and purification of DNA for 16S amplicon and metagenomic analysis, performed data analysis of 16S amplicons and metagenomes, interpretation of data, and drafting the paper. XD: Conducted metagenomic data analysis which included processing of QC and assembly of reads, generating gene abundance, and phylogeny trees. AA: Participated in data analysis of 16S amplicons and drafting the paper. HP: Contributed to sample collections. MS: Contributed to metagenomic data analysis. GV: Contributed to design of the study, interpretation of data, drafting the paper, final approval of the version to be published.

### Conflict of interest statement

The authors declare that the research was conducted in the absence of any commercial or financial relationships that could be construed as a potential conflict of interest.
