# Supplementary material for: Metagenomic Analysis Indicates Epsilonproteobacteria as a Potential Cause of Microbial Corrosion in Pipelines Injected with Bisulfite
Source: Front Microbiol. 2016 Jan 28;7:28. doi: 10.3389/fmicb.2016.00028 (PMC4729907; doi:10.3389/fmicb.2016.00028)
Supplement: Supplementary file 1 [file Table1.DOCX]

**Table S1| Results of chemical analysis of pipe-associated water (PAW) and pipe- associate solids (PAS) upstream (616P) or downstream (821TP) from the SBS injection point (Park *et al*., 2011).**
