# Supplementary material for: Metagenomic Analysis Indicates Epsilonproteobacteria as a Potential Cause of Microbial Corrosion in Pipelines Injected with Bisulfite
Source: Front Microbiol. 2016 Jan 28;7:28. doi: 10.3389/fmicb.2016.00028 (PMC4729907; doi:10.3389/fmicb.2016.00028)
Supplement: Supplementary file 3 [file Table3.DOCX]

**Table S3| Distribution and abundance of contigs of clades from the PAS-616P and PAS-821TP metagenomes on the phylogenetic trees of *hynL* genes for [NiFe]-hydrogenase shown in Figures 2 and 3.** Abundance is relative to the numbers of *rpoB* genes, as in Table 3.
