# Supplementary material for: Metagenomic Analysis Indicates Epsilonproteobacteria as a Potential Cause of Microbial Corrosion in Pipelines Injected with Bisulfite
Source: Front Microbiol. 2016 Jan 28;7:28. doi: 10.3389/fmicb.2016.00028 (PMC4729907; doi:10.3389/fmicb.2016.00028)
Supplement: Supplementary file 3 [file Table2.docx]

	PAS-616P		PAS-821TP	
Taxon term (order;family;genu)	16S amp	454 16S	16S amp	454 16S
Methanobacteriales;Methanobacteriaceae;uncultured	**68.54**	**24.26**	**48.24**	**3.41**
Methanomicrobiales;Methanocalculaceae; Methanocalculus	0.05	0	0.01	0
Methanomicrobiales;Methanomicrobiaceae;Methanoculleus	0.01	0	0	0
Methanosarcinales;Methanosaetaceae;Methanosaeta	0.03	0	0	0
Methanosarcinales;Methanosarcinaceae;Methanolobus	**6.35**	**3.19**	**1.61**	0
Methanosarcinales;Methanosarcinaceae;Methanosarcina	0.02	0.21	0	0
Desulfobacterales;Desulfobacteraceae;Desulfobacterium	0.03	0	0.04	0
Desulfobacterales;Desulfobacteraceae;Desulfobacula	0	0	0.05	0
Desulfobacterales;Desulfobacteraceae;Desulfotignum	0.03	0	0	0
Desulfobacterales;Desulfobulbaceae	0.10	1.06	0.47	1.56
Desulfobacterales;Desulfobulbaceae;Desulfobulbus	0.02	0.21	0.02	0
Desulfobacterales;Desulfobulbaceae;Desulfocapsa	**5.26**	**13.40**	**16.32**	**21.76**
Desulfobacterales;Desulfobulbaceae;Desulfurivibrio	0.07	2.55	0.01	0.00
Desulfovibrionales;Desulfomicrobiaceae;Desulfomicrobium	**14.06**	**40.21**	**28.89**	**49.36**
Desulfovibrionales;Desulfovibrionaceae;Desulfovibrio	0.05	0	0.04	0.14
Desulfuromonadales;Desulfuromonadaceae;Desulfuromonas	0.14	0	0.01	0
Campylobacterales	0	0	0	0
Campylobacterales;Campylobacteraceae;Arcobacter	0.01	0.21	0	0
Campylobacterales;Campylobacteraceae;Sulfurospirillum	0.01	0	0	0
Campylobacterales;Helicobacteraceae;Sulfuricurvum	0	0.43	0.19	**4.41**
Campylobacterales;Helicobacteraceae;Sulfurovum	0	0	0	0.85
Sum	94.76	85.73	95.90	81.63
